# Health Management of an HIV Testing and Counseling Center: Nursing Contributions

**DOI:** 10.1590/0034-7167-2023-0217

**Published:** 2024-03-15

**Authors:** Patrícia dos Santos Augusto, Camila Pureza Guimarães da Silva, Tânia Cristina Franco Santos, Lilian Dias Ennes, Carolina Fraga Paiva, Antonio José de Almeida

**Affiliations:** I Maternidade Municipal Fernando Magalhães. Rio de Janeiro, Rio de Janeiro, Brazil; II Universidade Federal do Rio de Janeiro. Rio de Janeiro, Rio de Janeiro, Brazil

**Keywords:** HIV, History of Nursing, Counseling, Health Policy, Nursing, VIH, Historia de la Enfermería, Consejo, Política de Salud, Enfermería, HIV, História da Enfermagem, Aconselhamento, Política de Saúde, Enfermagem

## Abstract

**Objectives:**

to analyze the role of nursing in the establishment of an HIV/AIDS Testing and Counseling Center in a Brazilian municipality.

**Methods:**

a historical study utilizing primary sources, including documents and oral accounts, involving a total of ten participants. The study encompasses the years 1997 and 1998. The research took place at the Testing and Counseling Center in São João de Meriti. Data was collected from March to May 2022.

**Results:**

nursing made significant contributions through the development of training initiatives led by nurses, who were also responsible for individual and group counseling, as well as HIV testing requests.

**Final Considerations:**

nursing played a crucial role in the center and in the management of Sexually Transmitted Infections, being involved in all stages of treatment in accordance with current public health policy.

## RESUMO

**Objetivos:**

analisar a participação da enfermagem no processo de criação do Centro de Testagem e Aconselhamento para pessoas com HIV/Aids de um município brasileiro.

**Métodos:**

estudo histórico com fontes diretas constituídas por documentos e fontes orais, totalizando dez participantes. O recorte compreende os anos de 1997 e 1998. O cenário de estudo foi o Centro de Testagem e Aconselhamento de São João de Meriti. Os dados foram coletados de março a maio de 2022.

**Resultados:**

a enfermagem contribuiu por meio do desenvolvimento de ações de capacitação pelo enfermeiro, que também era responsável pelas atividades de aconselhamento individual e coletivo, além da solicitação de exame anti-HIV**. Considerações Finais**: a enfermagem representou uma categoria responsável por ações relevantes no centro e no controle das Infecções Sexualmente Transmissíveis, estando presente em todas as etapas do tratamento em consonância com a política pública de saúde vigente.

## RESUMEN

**Objetivos:**

analizar la participación de la enfermería en el proceso de creación del Centro de Pruebas y Consejería para personas con VIH/Sida de un municipio brasileño.

**Métodos:**

estudio histórico con fuentes directas constituidas por documentos y fuentes orales, sumando diez participantes. El periodo abarca los años 1997 y 1998. El escenario del estudio fue el Centro de Pruebas y Consejería de São João de Meriti. Los datos se recolectaron de marzo a mayo de 2022.

**Resultados:**

la enfermería contribuyó mediante el desarrollo de acciones de capacitación por parte del enfermero, que también era responsable de las actividades de asesoramiento individual y colectivo, además de la solicitud de exámenes anti-VIH.

**Consideraciones Finales:**

la enfermería representó una categoría responsable por acciones relevantes en el centro y en el control de las Infecciones de Transmisión Sexual (ITS), estando presente en todas las etapas del tratamiento en consonancia con la política pública de salud vigente.

## INTRODUCTION

The HIV pandemic is currently a significant public health concern, with 1.5 million new infections and 650,000 deaths annually^(1)^. Recent studies indicate that the persistence of detectable risk factors for over twenty years emphasizes the importance of research on this topic^(2-3)^, which has evolved over time and reshaped the healthcare landscape.

The historiography of health has emphasized that the emergence of the disease in the 1980s was a global milestone in the healthcare field. The first official report of immunodeficiency cases was issued on June 5, 1981, in the “Morbidity and Mortality Weekly Report” (MMWR) by the Centers for Disease Control and Prevention (CDC) in the United States, head quartered in Atlanta, Georgia^(4-5)^.

While conducting a historical search for Acquired Immunodeficiency Syndrome (AIDS) in Brazil, it was observed that the disease was detected early in the country, as Brazilian doctors closely monitored information about the new ailment. The first Brazilian case occurred in the state of São Paulo in September 1982 when the patient presented a lesion on the right foot, and the diagnosis established through a biopsy was Kaposi’s sarcoma^(4)^.

By the end of 1983, AIDS had become a reality in various Brazilian cities. It was impossible to overlook, and as it had evolved into an epidemic, the Brazilian press frequently covered news about the new disease^(6)^.

From that point onward, AIDS became a subject in the mainstream press and the so-called sensationalist press. Throughout 1983, both types of media published reports that referred to AIDS as the “disease affecting homosexuals”, “gay cancer”, or “gay plague”, terms also used in the American press. Some publications featured exaggerated news that induced panic among the population, while others adopted a moralistic and discriminatory tone toward the most affected groups at the time^(6)^.

However, over the course of the AIDS epidemic in Brazil, there were significant changes in the affected population. Initially, the disease primarily affected a specific group of people, namely homosexual men in major urban centers, considered to be of higher social status. However, starting in 1983, cases of the disease began to occur in individuals outside of what was considered the “risk group”. The epidemic assumed new characteristics, affecting the population more broadly, with an increase in cases among heterosexuals, women, young individuals, children, and people of lower social status^(7)^.

In response to the HIV/AIDS epidemic, one of the initiatives involved establishing Anonymous Testing Centers/Testing and Counseling Center (TCC). The primary actions included confidential and anonymous provision of anti-HIV serological testing, as well as health education and counseling for all individuals seeking the service^(8)^.

This milestone was of utmost importance in combating the AIDS epidemic. Initially named Anonymous Testing Centers, they were later rebranded as Centers for Support and Serological Counseling (COAS). Their strategic guidelines for implementation aimed to establish partnerships with State and Municipal Health Departments, especially in cities of significant epidemiological importance. In 1997, the National Coordination of Sexually Transmitted Diseases/AIDS, renamed them as Testing and Counseling Centers, reflecting the principles guiding the integration and consolidation of these services^(9)^.

It should be noted that, for the integration and consolidation of TCC as a Federal Government initiative, an international cooperation agreement was necessary, focusing on the control of Sexually Transmitted Infections (STIs) and AIDS. This investment was made through the AIDS-I Project, which represented a partnership between the Brazilian government and the World Bank and included measures for promoting, protecting, and preventing HIV. Once the AIDS-I Project was implemented, the necessary financial resources for establishing TCC became available. Priority was given to regions with a higher epidemiological impact of the disease in agreements with State and Municipal Health Departments^(4-10)^.

In this manner, the creation of the Testing and Counseling Center of São João de Meriti (SJM) began under the auspices of the municipality and the Municipal Health Department of this city, with active participation from the Coordinator of Programs and Community Health of the Department of Collective Health, then under the responsibility of a nurse. To facilitate this coordination, a project was structured to select human resources, provide training for these professionals, and determine a suitable location for TCC’s operation, with active involvement of nursing throughout these stages.

This study aims to contribute to a better understanding of the HIV/AIDS epidemic and the strategies for creating a specialized service with a significant social impact in the fight against the disease in Brazil, particularly in the municipality of São João de Meriti.

To achieve this purpose, the following guiding question was formulated: How did nursing contribute to the process of creating the Testing and Counseling Center for people with HIV/AIDS in the municipality of São João de Meriti, in the state of Rio de Janeiro, Brazil?

## OBJECTIVES

To analyze the role of nursing in the establishment of an HIV/AIDS Testing and Counseling Center in a Brazilian municipality.

## METHODS

### Ethical aspects

Ethical considerations were adhered to following the recommendations of Resolutions No. 466/12 and 510/16 of the National Health Council. For interviews, all participants signed an Informed Consent Form. After transcription, these transcripts were provided to each participant for reading and validation. Access to and consultation of written documents were authorized by municipal authorities after signing an assent form. The study was approved by the Research Ethics Committee for Human Subjects of the institution.

### Study type

This is a historical approach study, from the perspective of Contemporary History, with its corpus consisting of direct written and oral historical sources, and indirect sources used to support the discussion of results. The guidelines from the guide for writing qualitative research reports (COREQ) were followed. The temporal scope covers the years 1997 and 1998, the initial period of actions for the creation of this Testing and Counseling Center.

### Study setting

The setting is the Testing and Counseling Center, a reference unit for HIV/AIDS prevention and diagnosis actions in the municipality of São João de Meriti, in the state of Rio de Janeiro.

It is important to note that the municipality of São João de Meriti is located in the Baixada Fluminense, Metropolitan Region I of the state of Rio de Janeiro. The Baixada Fluminense consists of 13 municipalities: Belford Roxo, Duque de Caxias, Guapimirim, Itaguaí, Japeri, Magé, Mesquita, Nilópolis, Nova Iguaçu, Paracambi, Queimados, São João de Meriti and Seropédica^(11)^.

According to research by the Brazilian Institute of Geography and Statistics (IBGE), conducted within the temporal scope of this study, the municipality of São João de Meriti had an estimated population of 458,673 inhabitants, covering a territorial area of 35,216 square kilometers (km^2^). Furthermore, it had a Human Development Index (HDI) of 0.719, below the national average. Today, the city has the highest population density in Brazil and Latin America, with approximately 13,000 inhabitants per km^2^, a characteristic that earned it the nickname “Ant Farm of the Americas”^(11)^.

### Data collection and organization

The direct written historical sources consisted of regulations, manuals from the Ministry of Health, and the Project for the Creation of the Testing and Counseling Center in the municipality of São João de Meriti.

The direct oral sources comprised interviews with a total of ten participants, including: physicians (2); nurses (1); psychologists (1); social workers (2); nursing assistants (2); administrative staff (2). The interviewees held positions and roles such as Coordinator of Community Health Programs, coordinator, and technical manager of TCC/SJM, counselors, collectors, and receptionists, and they were involved in the process of creating TCC/SJM.

For the interviews, a semi-structured script with questions about the planning for the creation of TCC/SJM was used, including topics such as: selection and training of professionals; defining the location and its organization; challenges and strategies for overcoming them.

The interviews lasted from 30 to 120 minutes and were audio-recorded. They were conducted by the first author of the study from March to May 2022, at the participants’ workplace, reserved by TCC/SJM. To ensure anonymity, the participants were identified by the letter P followed by a cardinal number in the order of the interviews (P01…P10). The selection of participants was based on the nominal list of employees from that time.

Participants who joined the Municipal STI/AIDS Program and TCC/SJM after the temporal scope of this research were excluded from the study, as well as participants who could not be located or did not wish to participate in the research.

The indirect sources consisted of scientific articles on the topic.

### Data analysis

For the analysis of the documentary corpus, the written sources were cataloged in chronological order. Subsequently, external and internal criticism was performed to ensure the authenticity, legitimacy, accuracy, and reliability of the documents^(12)^. Regarding the direct oral sources, the interviews were transcribed and validated by the participants^(13)^. From then on, the documentary corpus underwent active questioning of the documents, adopting an independent stance from the official version, which allowed for a better understanding of the historical phenomenon. The reliability of the results was ensured by valuing the documentary set as a whole, rather than individual documents, with a focus on the following category, according to the thematic analysis proposed by Minayo^(14)^: the location for the operation of the Testing and Counseling Center, and the process of selection and training of the team’s professionals.

## RESULTS

### Location for the Operation of the Testing and Counseling Center

The organizational principles of the Regulations for the Organization and Operation of Serological Counseling and Support Centers (NOFCOAS), regarding accessibility, stipulated that Testing and Counseling Centers should be situated in easily accessible locations for the general population and target populations. Additionally, TCCs could conduct their activities within the premises of existing Health Units that served as a health reference for the local population, including the possibility of outpatient clinics for the treatment of STIs/AIDS. There was also the option of having their own physical facilities. The decision on this matter would be left to local coordinators, based on the evaluation of each situation^(15)^.

To comply with the accessibility principle in defining a location for the creation of TCC/SJM, as outlined in the Ministry of Health’s Regulations for Organization and Operation, the Coordinator of Community Health Programs in SJM needed to identify a suitable location for the service.

The chosen location to accommodate TCC/SJM was the Aníbal Viriato de Azevedo Health Center, a reference unit for outpatient care of cases of tuberculosis, leprosy, and patients with reactive serology for HIV and AIDS amenable to outpatient management. This Health Center was situated in the Vilar dos Teles District, where the municipal administrative center was located. Its location was easily accessible due to its proximity to various municipal and intermunicipal bus lines, as indicated in the participants’ statements in the study:

[...] *there was already a center for diseases linked to the Collective Health Program. And there was a larger physical space; it was considered a geographical matter, easy for everyone to access initially. It’s a place where a person could take just one bus; from some places, you could even walk, so that was considered. And the physical area was very good.* [...]. (P04)

[...] *Aníbal* [Aníbal Viriato de Azevedo Health Center] *was the largest health center; it was one of the biggest clinics there was, and because it’s next to the city hall, I think it was a political vision because it’s right in front of the city hall square* [...]. (P10)

To choose the location for the operation of TCC, it was necessary to observe certain principles established by the Regulations for the Organization and Operation of Serological Counseling and Support Centers. Among these, the installation of the Center within the premises of an existing health unit and accessibility to users stood out, ensuring easy access for those seeking assistance^(10)^.

The National Program for the Control of STIs/AIDS of the Ministry of Health (MS) established criteria for the creation of TCCs, such as the size of the population, the epidemiological profile, and the existence of an STIs/AIDS program in the municipality. In 1997, actions began for the creation of the Testing and Counseling Center in the municipality of São João de Meriti (TCC/SJM). This task was the responsibility of the Coordinator of Community Health Programs, linked to the Department of Collective Health of the Municipal Health Secretariat (SMS), as illustrated in the following excerpt from an interview:

[...] *I was the Coordinator of Community Health Programs at the State Department of Health at the time. The Ministry of Health at the time decided to increase the number of testing and counseling centers throughout the country. And what they did was choose municipalities that met certain criteria: population criteria, epidemiological profile, and the existence of an AIDS program already in the municipality* [...]. (P01)

The first initiative of the Coordinator of Community Health Programs was the development of a project for the authorization of the creation of TCC in the municipality of São João de Meriti, to be submitted and approved by the Ministry of Health. To prepare this project, training was required, provided by professionals from the Ministry of Health. The investment in this process is highlighted in the testimony of one of the study participants:

[...] *the Ministry of Health brought together the Coordinators of Community Health and Programs to Brasília, so I went to Brasília to be trained to develop the project* [...]. (P01)

The Project for the Establishment of TCC/SJM followed the Regulations for the Organization and Operation of the Testing and Counseling Center of the National Program for the Control of Sexually Transmitted Diseases and AIDS. These regulations stipulated that the TCC would require a minimum team to operate, as well as the requirements that the team be interdisciplinary and trained to serve users, and that a suitable location be provided for its installation.

### Process of Selection of Team Professionals

According to the Regulations for the Organization and Operation of Testing and Counseling Centers and the Implementation Project of TCC/COAS in the Municipality of São João de Meriti, the minimum team should consist of four counselors - including physicians, nurses, mental health professionals, social workers, or educators - in addition to other professionals with a higher education degree in the healthcare field, two collectors, two receptionists, and cleaning staff. The team, diverse and interdisciplinary, should have a representative from each category^(15)^.

Team members held commissioned positions in the municipality of São João de Meriti. Some were invited by the Coordinator of the Community Health Program of São João de Meriti, while others applied for the positions, as noted in the testimonials:

[...] *the selection process was gradual, most were commissioned employees, those who were interested in working came and stayed. It was people’s choice; we had no difficulty in forming the team* [...]. (P02)

[...] *I worked with the mayor, and I was offered to be part of this group* [TCC interdisciplinary team]*, and they joined the government. I introduced myself at the Program Department, at the coordination. The next day they had decided that I would go to the IST/AIDS program* [...]. (P10)

To ensure quality service in these centers, it was crucial to form a diverse team, including physicians, nurses, psychologists, social workers, receptionists, and blood collectors. The composition of the interdisciplinary team selected to work at CTT/SJM at the time of its creation is illustrated in the following excerpt from an interview:

[...] *at the beginning, there were two of us at the front desk* [receptionists], *we had two psychologists, two social workers, the nursing technicians* [handled the collection], *we had one doctor, who was the coordinator, and there was the nurse* [...]. (P09)

The nurse stood out as a crucial professional, considering that a significant portion of the actions at the CTA was carried out by these professionals, as emphasized in the participants’ accounts. The importance of this professional is evident in the following statement:

[...] *they were essential because the nurse, he was a professor, so he was great at training us, he trained many professionals because he had great didactics, so he provided training, he took care of biosafety, blood, he received patients with HIV* [...]. (P03)

[...] *the nurse started working as a counselor, doing tests, those things. He started working directly with HIV patients. The first consultation was with the nurse; he would provide guidance on the disease, he would request viral load, CD4, and other tests* [...]. (P08)

The number of professionals was determined according to the demand of the TCC. Among the team members, the nurse was responsible for individual and group counseling, as well as for requesting the anti-HIV test. Nursing technicians, as in other TCC, were responsible for blood collection^(10,15)^.

[...] *I would talk to the patients first, and then I would collect material. We would discuss the entire procedure that would be performed on the patient, that whole thing about “look, this syringe and this needle here are disposable”* [...]. (P06)

[...] *the nursing assistants were in charge of blood collection. They would check the patients’ weight and measurements* [...]. (P07)

In addition to the quantity of professionals in the interdisciplinary team, it was essential that all of them were adequately trained and qualified to serve the center’s users.

### Training Process for Team Professionals

Health professionals should be capable of providing support in counseling for STIs/HIV/AIDS, addressing not only the biophysical aspects but also emotional, family, social, cultural, and political issues^(8,16)^.

The methodologies for STI/HIV/AIDS counseling training should be fundamentally participatory, allowing professionals to retrieve and enhance their skills in managing the affective-emotional aspects present in the user-assistance relationship. In addition to expositional-dialogue sessions, these training programs should encompass group dynamics, awareness and experiential workshops, and techniques for expressing emotions^(16-17)^.

In accordance with the protocols and guidelines of the National Program for the Control of STIs/HIV/AIDS coordination, TCC/SJM health professionals underwent training, as indicated in the interview excerpts:

[...] *the training was continuous, almost to the point of excess. I attended all the training sessions, short courses with up to 60 hours of duration; I have over 60 certificates in my folder. Courses were happening all the time, I traveled, participated in conferences, and exchanges. I took some courses at São Francisco* [Hospital Escola São Francisco de Assis (HESFA)] [...]. (P04)

[...] *the first training was at São Francisco de Assis Hospital* [Hospital Escola São Francisco de Assis (HESFA)], *all team members went for training, and then the TCC* [TCC/SJM] *opened here. We also went to São Paulo for training* [...]. (P05)

It’s important to emphasize that at the time of TCC/SJM’s creation, approximately ten years after the onset of the AIDS epidemic in Brazil, the etiological agent and modes of virus transmission were already known. However, not everyone had access to this information, which meant that AIDS continued to be a challenge for some healthcare professionals. This also contributed to the stigma associated with individuals living with HIV/AIDS and healthcare professionals working with diagnosed patients.

Regarding the stigma faced by healthcare professionals due to the lack of information, HIV/AIDS, being an incurable and potentially fatal disease, posed a threat to both the patient and their families, as well as to healthcare professionals. This situation was exacerbated by the scarcity and distorted, stigmatized transmission of information about the disease^(4,18)^:

[...] *at that time, no one wanted to work with HIV patients; no one wanted to work in testing or the AIDS program. When they talked about the AIDS program, no employee wanted to come. When they invited me, they already offered me a proposal to earn a little more, and that’s how I came here* [...]. (P09)

In addition to these challenges, the reluctant interest in working with HIV/AIDS was influenced by the stigma directed at professionals working in this sector, with the mistaken belief that they chose to work in this area because they were also carriers of the disease, as evidenced in interview excerpts:

[...] *and in terms of treatment, even we were discriminated against, they would say things like, “don’t go back there, that’s where the AIDS people are, they’re all contaminated”. They thought that because we worked there, we were also infected* [...]. (P05)

[...] *at that time, there was a lot of prejudice, TCC/SJM was set up to provide support for Baixada Fluminense because people here are very radical and prejudiced! Patients from other municipalities came here for treatment, and residents from here went to other municipalities out of fear of discrimination. One patient said his wall was graffiti when they found out he was HIV positive* [...]. (P06)

Just as an interdisciplinary team trained to serve users was necessary, the importance of a suitable location for the center’s installation was equally crucial.

## DISCUSSION

The Ministry of Health recommended the establishment of Testing and Counseling Centers in easily accessible locations for the population, in facilities physically separate from other healthcare structures, and staffed by their own multiprofessional teams. The Ministry of Health also bore the responsibility for the necessary investments in physical infrastructure, equipment and furniture acquisition, and professional training. States and municipalities were tasked with maintaining the units, teams, and consumable materials, including the procurement of serological tests. The Ministry’s financing policy was implemented between 1994 and 1998, under the first Loan Agreement with the World Bank (AIDS I), aimed at strengthening the national response to the AIDS epidemic^(19)^.

TCC/SJM adhered to the Ministry of Health’s regulations, which specified that the technical team of the TCC should have dedicated facilities for conducting its activities, such as reception rooms, group session areas, individual counseling spaces, blood collection rooms, and archives. Services were provided free of charge and addressed both spontaneous requests and referrals from other services or healthcare professionals.

The Testing and Counseling Center in the municipality of São João de Meriti served as a privileged social space, comprised of social agents with various forms of social and cultural capital, concerning HIV/AIDS prevention and diagnosis.

In order to ensure proper care for the population of São João de Meriti in terms of HIV/AIDS prevention and diagnosis, the Coordinator of Community Health Programs, affiliated with the Department of Public Health of the Municipal Health Department, was responsible for actions to combat the epidemic and took charge of creating the TCC in the municipality.

To achieve this, various actions were necessary, and the Coordinator of Community Health Programs implemented strategies focused on establishing TCC/SJM, beginning with updating his knowledge about the infection and the disease by participating in different scientific events. These training sessions, organized by recognized professionals and researchers in the field of health, facilitated the accumulation of a significant amount of scientific and social capital, resulting from professional qualifications to lead the organization of the TCC.

After receiving training from the Ministry of Health, the Coordinator of Community Health Programs developed the project for the establishment of TCC/SJM, titled “Implementation of a Serological Counseling and Support Center in the Municipality of São João de Meriti”, in accordance with the guidelines of the Regulations for the Organization and Operation of the National Program for the Control of STIs/AIDS. As a result, he became the professional with the highest symbolic capital, positioning himself prominently for the coordination of TCC/SJM. Consequently, he served as an authorized spokesperson in the field of infectious diseases and within the interdisciplinary team of TCC/SJM.

Regarding the process of selecting professionals for the team to work at the TCC in the municipality of São João de Meriti, it occurred gradually, respecting its interdisciplinary nature. All team professionals who worked at TCC/SJM participated in and shared all aspects of the care process, adhering to the specific competencies of each professional category. It is worth noting that HIV/AIDS, as a chronic disease, presents challenges to healthcare professionals that extend beyond actions aimed solely at containing the epidemic. Therefore, actions directed towards treatment and knowledge dissemination are crucial to prevent discrimination and the proliferation of social stigma^(20-21)^.

In this context, an individual displaying clinical signs of AIDS or the presence of HIV was considered to possess a stigmatizing attribute, leading to social marginalization and victimization due to prejudice. This judgment applied both to the formation of their actual social identity, as evidenced by classic AIDS symptoms, and to their virtual social identity, resulting from infection without clinical manifestation^(21)^.

Recent studies indicate that stigma is more pronounced in less developed countries, highlighting the responsibility of nations and health organizations to focus efforts on educating the population about prevention and promoting positive portrayals of individuals living with HIV/AIDS. This contributes to a society whose social values are rooted in the human rights of all individuals with illnesses. Additionally, healthcare professionals can play a role in destigmatizing this condition by investing in training programs that address the historical, cultural, socioeconomic, and political dimensions of the epidemic, in addition to biomedical interventions^(22-23)^.

Regarding the training process for team professionals, according to the research, there was a significant investment by the coordinator in the training of the TCC/SJM team. Training sessions were conducted both on-site, within the physical space of the TCC, and externally at the TCC/HESFA/UFRJ, which was considered the national reference TCC for training.

The training aimed to empower professionals through reflective and collaborative education, understood as a dynamic and ongoing process of knowledge construction through free thinking and critical-reflexive awareness. Its goal was to develop a personal and professional commitment to transforming reality^(24-25)^.

As such, the training and capacity-building efforts aimed to enhance the roles of each professional and also to analyze and evaluate the process so that professionals felt confident in performing the procedures^(23)^. Building trust through training among team members was essential for achieving change, overcoming challenges, promoting professional growth, personal development, and vital strategies and organization for quality improvement.

It is worth noting that the various forms of training for the TCC/SJM professional team contributed to the formation of symbolic capital, expanding the team’s knowledge and providing updates to their expertise. This condition is characterized by the innovation of specific knowledge in the context of HIV/AIDS, impacting the reconfiguration of care for people living with HIV/AIDS in the municipality of SJM.

Nursing played a crucial role in the creation, development, and operation of TCC/SJM, as was the case in other similar experiences. Nursing professionals were responsible for many actions in TCCs and in the control of STIs/AIDS because they are present in all stages of HIV treatment, from diagnosis. Therefore, the role of nursing in this scenario was extremely relevant^(10,15-16)^.

The nurse stood out as a crucial professional, carrying out a significant portion of the actions in TCCs. This importance underscores recognition by managers and the multidisciplinary team. In the context of TCC/SJM, the nurse conducted training sessions, contributing to the team’s scientific capital updates. Additionally, the nurse actively participated in the entire process involving diagnosis, treatment, and disease control, being responsible for individual and group counseling sessions and requesting anti-HIV tests. Nursing technicians were involved in blood collection, providing support to the nurse.

In this sense, the coordinator, the interdisciplinary team, and especially nursing contributed to ensuring that the TCC creation process met the requirements established by the Ministry of Health’s National Program for the Control of STIs/AIDS, making it a well-established and early diagnostic space for HIV/AIDS in São João de Meriti.

### Study limitations

The limitations of this study are related to the possibility of finding additional historical sources. Despite the comprehensive and thorough search for historical sources in this research, identifying additional documents in future studies could lead to adjustments in this historical narrative.

### Contributions to Nursing, Health, and Public Policy

The contribution of this study can help in understanding the context and the establishment of a TCC, as well as its impact on the prevention of HIV/AIDS infection. In the healthcare context, it highlights the strategies employed by healthcare professionals in the fight against the epidemic in Brazil and São João de Meriti, through the accumulation of knowledge about the health needs of the population and public policies, resulting in the establishment of a Testing and Counseling Center.

In this manner, this work also contributes to expanding the knowledge of those working in this field, with the goal of promoting a better comprehension and adoption of this approach, with a focus on the quality of healthcare at all levels. This research also serves as an important analytical tool for enhancing the understanding of the trajectory of nursing in this mode of care in the country.

## FINAL CONSIDERATIONS

With the development of the project for the São João de Meriti Testing and Counseling Center (TCC/SJM), essential actions began, such as the selection process for the team professionals, their training, and the determination of an appropriate location for the TCC’s operation. These challenges were particularly pronounced due to the clientele being HIV/AIDS patients, a disease that was little known in the field of health at the time, with no cure, high mortality rates, and a strong stigma.

Stigmatization manifested in various ways. On one hand, there was a lack of knowledge among healthcare professionals, which generated apprehension about working in the TCC. On the other hand, there was stigma directed towards people with HIV/AIDS and healthcare professionals working with these patients, especially nursing professionals.

In this scenario, nursing took on a leading role and was widely recognized by managers. Their contribution was essential in the creation, development, and operation of TCC/SJM. Nursing represented a category responsible for significant actions in the center and in the control of STD/AIDS, playing a role in all stages of HIV/AIDS treatment, in line with prevailing public health policy.
